# New Behavioral Signs of Consciousness in Patients with Severe Brain Injuries

**DOI:** 10.1055/a-1883-0861

**Published:** 2022-09-13

**Authors:** Beril Mat, Leandro R.D. Sanz, Anat Arzi, Melanie Boly, Steven Laureys, Olivia Gosseries

**Affiliations:** 1 Coma Science Group, GIGA Consciousness, University of Liège, Liège, Belgium; 2 Department of Neurology, University of Wisconsin–Madison, Madison, Wisconsin; 3 Centre du Cerveau2, University Hospital of Liège, Liège, Belgium; 4 Paris Brain Institute, Paris, France; 5 Department of Medical Neurobiology and Cognitive Sciences, The Hebrew University of Jerusalem, Jerusalem, Israel; 6 Department of Psychiatry, University of Wisconsin–Madison, Madison, Wisconsin; 7 Joint International Research Unit on Consciousness, CERVO Brain Research Centre, CIUSS, Laval University, Québec, Canada

**Keywords:** disorders of consciousness, behavioral assessment, signs of consciousness, postcomatose state

## Abstract

Diagnostic and prognostic assessment of patients with disorders of consciousness (DoC) presents ethical and clinical implications as they may affect the course of medical treatment and the decision to withdraw life-sustaining therapy. There has been increasing research in this field to lower misdiagnosis rates by developing standardized and consensual tools to detect consciousness. In this article, we summarize recent evidence regarding behavioral signs that are not yet included in the current clinical guidelines but could detect consciousness. The new potential behavioral signs of consciousness described here are as follows: resistance to eye opening, spontaneous eye blink rate, auditory localization, habituation of auditory startle reflex, olfactory sniffing, efficacy of swallowing/oral feeding, leg crossing, facial expressions to noxious stimulation, and subtle motor behaviors. All of these signs show promising results in discriminating patients' level of consciousness. Multimodal studies with large sample sizes in different centers are needed to further evaluate whether these behaviors reliably indicate the presence of consciousness. Future translation of these research findings into clinical practice has potential to improve the accuracy of diagnosis and prognostication for patients with DoC.

Advances in medical knowledge and technologies have made it possible for many patients who had traumatic or nontraumatic severe brain injuries to survive, and, consequently, transition into or remain in what are now referred to as disorders of consciousness (DoC). Over one million people are affected by DoC each year worldwide. 1 2 Among DoC patients, withdrawal of life-sustaining therapy is a frequent cause of death. 3 4 5 6 However, the lack of knowledge about patients' consciousness level and their potential for long-term recovery remains a challenge throughout this decision-making process. Thus, correct diagnosis and outcome prediction of this vulnerable patient population have tremendous clinical and ethical implications for patients, their caregivers, and clinicians.

DoC are categorized on a spectrum of diagnostic entities, ranging from the most severe state, coma, to vegetative state/unresponsive wakefulness syndrome (VS/UWS), minimally conscious state minus (MCS − ), minimally conscious state plus (MCS + ), and emergence from MCS (eMCS). The acute period of DoC is defined as the first 28 days after the brain injury, and subacute-to-chronic (or “prolonged” DoC) as longer than 28 days. 7 Coma is defined as the complete absence of arousal and awareness. 8 VS/UWS is defined as preserved arousal (eye opening spontaneously or upon stimulation) without awareness, the patient showing only reflexive behaviors. 9 10 11 MCS is defined as minimal, reproducible but inconsistent behavioral signs of awareness. 12 MCS− patients show nonreflex movements such as localization of noxious stimuli, visual pursuit or fixation, localization of objects, and movement or affective behaviors in a contextual manner to relevant environmental stimuli. MCS+ patients display behaviors related to language expression and comprehension, including command-following, intelligible verbalization, and intentional communication. 13 When patients demonstrate functional object use or functional communication, they are considered eMCS. 12 Cognitive motor dissociation (CMD), 14 functional locked-in syndrome, 13 MCS*, 15 16 and covert cognition 17 are terms suggested by different research teams to define behaviorally unresponsive patients who show brain activity compatible with (minimal) consciousness detected by functional magnetic resonance imaging (fMRI), electroencephalography (EEG), or positron emission tomography (PET). CMD is specifically used for patients who show no (VS/UWS) or little (MCS − ) behavioral evidence of consciousness at the bedside but have cortical responses related to language processing in fMRI or EEG active paradigms. 14 MCS* encompasses VS/UWS patients with CMD as well as VS/UWS patients who have residual brain activation in neuroimaging compatible with diagnosis of MCS even in the absence of active paradigms. 15 16

Accurate diagnosis of DoC patients is highly challenging. Indeed, over one-third of DoC patients previously diagnosed with VS/UWS by clinical consensus (based on behavioral observations and clinical experience) had evidence of consciousness when they were later evaluated based on standardized behavioral assessments using the Coma Recovery Scale-Revised (CRS-R). 18 19 20 However, it is important to note that, if performed only once, standardized behavioral assessment with the CRS-R can also lead to a 35% rate of misdiagnosis (compared with five CRS-R assessments). 21 Thus, repeated assessment (at least five times in a short period, e.g., 10 days) is of critical importance. The high misdiagnosis rates in DoC patients may be related to the lack of a proper gold standard to assess the presence of consciousness, and the need to integrate neuroimaging and new potential behavioral signs of consciousness into diagnostic guidelines. All these warrant the urgent need for improvement in diagnostic methods.

Currently available standardized behavioral scales to assess patients with DoC include, among others, the Glasgow Coma Scale (GCS), 8 the Full Outline of UnResponsiveness, 22 the Simplified Evaluation of CONsciousness Disorders (SECONDs), 23 24 and the CRS-R. 25 The American Congress of Rehabilitation Medicine Task Force and European Academy of Neurology recommends the use of repeated CRS-R in the assessment of patients with subacute-to-chronic DoC. 26 27

The CRS-R consists of 23 items composed of six subscales assessing auditory, visual, motor, oromotor/verbal functions, communication, and arousal. Among these items, 11 indicate an MCS diagnosis (six for MCS− and five for MCS + ). From these items, visual pursuit, reproducible command-following, and automatic motor response (e.g., nose scratching, grasping bedrail, grabbing tubes) were found to be the first three most common signs of MCS to reemerge after brain injury. 28 The five most frequently observed items detecting 99% of chronic MCS patients were visual fixation, visual pursuit, reproducible movement to command, automatic motor response, and localization to noxious stimulation. 29 When transitioning into MCS, chronic VS/UWS patients were found to show mostly only one behavioral sign (73%): visual fixation, visual pursuit, localization to noxious stimulation, reproducible movement to command, or functional communication. 30 Similarly, chronic MCS patients were also found to show mostly only one behavioral sign (64%) while transitioning into eMCS, either functional communication or functional object use 30 ( Fig. 1 ).

**Fig. 1:**
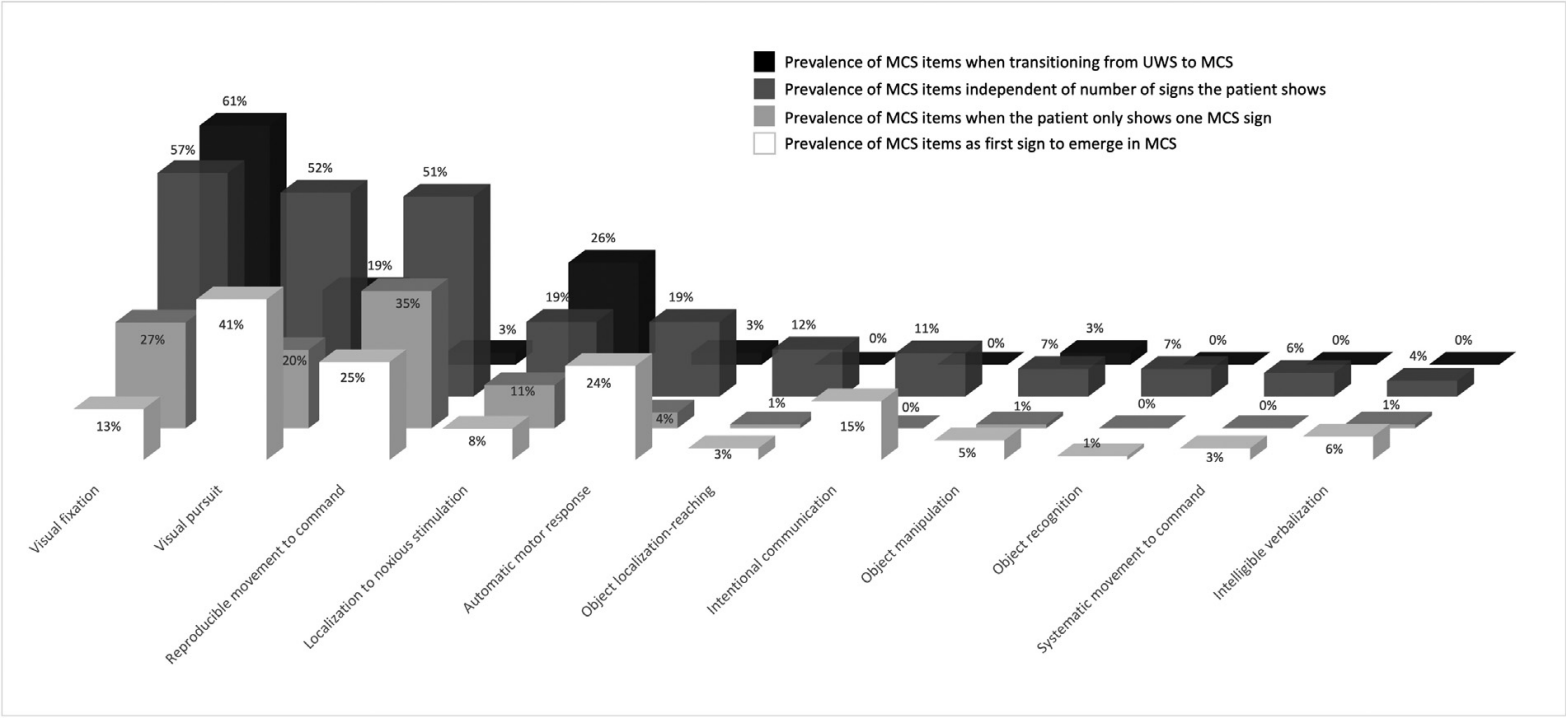
Prevalence of 11 minimally conscious state (MCS) items from the Coma Recovery Scale-Revised (CRS-R) in disorders of consciousness (DoC) patients from four different studies. **Black** , the prevalence of MCS items in the CRS-R in patients who transition from vegetative state/unresponsive wakefulness syndrome (VS/UWS) to MCS (adapted from Carrière et al 30 ). **Dark gray** , the prevalence of MCS items in the CRS-R taking into account all MCS items observed in the whole cohort of MCS patients, and ( **light gray** ) when taking into account MCS items observed in patients who show only one sign of consciousness (adapted from Wannez et al 29 ; in this study they included the first CRS-R where all MCS items were tested for every patient). In white, the frequency of CRS-R items is shown as temporally first ones to emerge in MCS patients (adapted from Martens et al 28 ; evidence of transition to consciousness was defined as 2 consecutive complete CRS-R within 7 days indicating new MCS or emergence from MCS). Given the prevalence of MCS items in this figure from different studies, we highly encourage paying attention to these five most prevalent items (visual fixation, visual pursuit, reproducible movement to command, automatic motor response, and localization to noxious stimulation).

In addition to the already available items that denote MCS in the CRS-R, recent studies suggest that other behaviors may be considered as signs of consciousness in DoC patients. The objective of this article is to summarize and review these new behavioral findings: resistance to eye opening, spontaneous eye blink rate, auditory localization, habituation of auditory startle reflex, olfactory sniffing, swallowing/oral feeding, facial expressions to noxious stimulation, subtle motor behavior assessed by Motor Behavioral Tool-revised (MBT-r), and leg crossing ( Table 1 and Fig. 2 ).

**Fig. 2:**
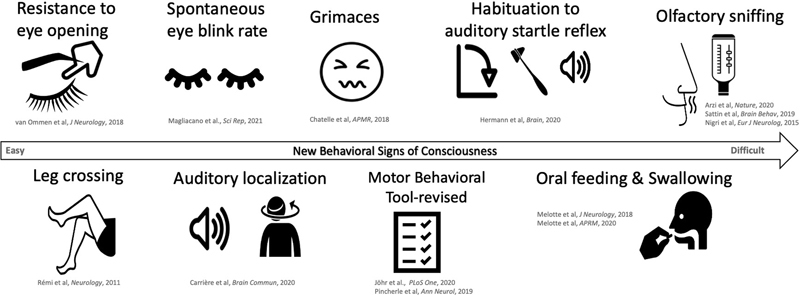
Potential new behavioral signs of consciousness. All the new behavioral signs of consciousness reviewed in this article have feasible, practical, and affordable assessment methods in clinical practice. In this illustration, the signs are displayed according to the complexity of the assessment and the need for expertise and/or equipment to carry out examinations. Depending on the resources of each center, we recommend using as many items as possible to improve the diagnostic accuracy.

**Table 1:** Summary of results from studies reviewed in this article

Author (year)	Behavior	No. of patients and sex	Age (mean±SD or median, IQR or range)	Onset (mean±SD or median, IQR or range)	Etiology	Behavioral tool	Diagnosis	Brain imaging	Main findings	Sign of C	Prognosis	Bias/Limitations
van Ommen et al 35 (2018)	Resistance to eye opening	79 36 F 43 M	37, IQR: 28–52	15 mo, IQR: 1–41 mo	42 TBI 25 post-anoxic encephalopathy due to cardiac arrest 6 stroke 5 SAH 2 encephalitis 1 hypoglycemia	CRS-R	23 VS/UWS 15 MCS− 41 MCS+	PET MRI	Significant relationship between REO and the level of consciousness. Higher REO repeatability in MCS. Atypical neuroimaging findings similar to MCS in VS/UWS patients with REO.	Yes	No correlation	Heterogeneity in time since onset, etiologies, and brain damage.
Magliacano et al 39 (2021)	Spontaneous eye blink rate	24 6 F 18 M	50 ± 18 VS/UWS 53 ± 18 MCS	6 ± 16 mo	7 TBI 9 ABI 8 vascular	CRS-R	10 VS/UWS 14 MCS	EEG	Eye blink rate was significantly higher in MCS patients compared with VS/UWS patients. A significant positive correlation was found between CRS-R index and eye blink rate.	Yes	n/a	Researcher who evaluated eye blink rates was not blinded to patients' diagnosis. Only investigating eye blink rate, but not other blink characteristics. Not recording all sessions for all patients at the same time of the day. Some patients were assessed less than 5 times by CRS-R for diagnosis. Small sample size.
Carrière et al 41 (2020)	Auditory localization	186 66 F 120 M	39 ± 16	9 mo, range: 1mo–29y	100 TBI 86 non-TBI	CRS-R	64 VS/UWS 28 MCS− 71 MCS+	PET fMRI hdEEG	Auditory localization increased with level of consciousness. Higher survival rates after 2-y follow-up in patients with auditory localization compared with patients without auditory localization. Higher fMRI functional connectivity between frontoparietal network and secondary occipital regions in VS/UWS patients with auditory localization. Higher participant coefficient in α band in VS/UWS patients with auditory localization.	Yes	Increased survival rate	Small sample size of VS/UWS with localization (auditory localization as the only “sign of consciousness” is rare). Subgroups not matched for age and time since injury. Missing clinical outcome data. Some patients had light sedation for fMRI. Lack of auditory evoked potentials or otoacoustic emissions to rule out deafness in the absence of auditory response.
Hermann et al 45 (2020)	Habituation to auditory startle reflex	96 M/F ratio: 1.8	44 ± 16	58 d, IQR: 31–236d	39 ABI 27 TBI 12 vascular 18 other	CRS-R	48 VS/UWS 48 MCS	PET DTI MRI hdEEG	More habituation in MCS compared with VS/UWS. Higher CRS-R scores in all subscales except communication in patients with habituation. Highest prevalence and sensitivity for habituation compared with the performance of all MCS items of the CRS-R to discriminate MCS. PET activity in salience and default mode networks correlated with habituation. Higher *θ* and *α* power, with higher prefrontal-temporal connectivity in patients with habituation. Higher recovery of command-following after 6-mo follow-up in VS/UWS with habituation.	Yes	Yes (recovery of command-following after six-mo follow up)	None reported.
Sattin et al 46 (2019)	Olfactory discrimination	11 6 F 5 M	57, IQR: 14	3–146 mo	6 ABI 3 hemorrhagic brain injury 1 ischemic and hemorrhagic brain injury 1 TBI + ABI	CRS-R	9 VS/UWS 2 MCS	fMRI	All MCS and 33% of VS/UWS had a discriminatory olfactory response. All VS/UWS with discriminatory olfactory response had olfactory-related activity in olfactory cortices in fMRI.	Yes	n/a	Small sample size. Lack of quantitative analysis of nasal airflow. Using only 4 odors could underestimate the real olfactory functions due to chance of specific anosmia. 4 patients excluded due to head movements in fMRI.
Nigri et al 47 (2016)	Olfactory processing	42 19 F 23 M *	57, IQR: 23–77 for VS/UWS; 44, IQR: 20–71 for MCS	26mo, IQR: 3–146 for VS/UWS; 41mo, IQR: 11–170 for MCS	17 ABI 7 hemorrhagic brain injury 6 TBI 2 ischemic and hemorrhagic brain injury 1 TBI + ABI	CRS-R	26 VS/UWS 7 MCS	fMRI	58% of VS/UWS, 100% of MCS showed odor-induced activity in primary olfactory areas. 39% of VS/UWS and 71% of MCS showed activation within a higher-order olfactory processing area. Most patients with anoxic brain injury had no activation in primary olfactory areas.	n/a	n/a	Small sample size. The excluded populations (due to movement in fMRI) were majorly MCS, and thus might have skewed the results.
Arzi et al 48 (2020)	Olfactory sniffing	43 8 F 35 M	43 ± 17	1mo–10mo	27 TBI 5 ABI 10 cerebrovascular accident 1 infection	CRS-R CNC	21 VS/UWS 22 MCS	n/a	Reduction of nasal airflow in response to odorants and empty jar presentation in MCS sessions, but not in VS/UWS sessions. Sniff response had 64.5% sensitivity to determine MCS. On individual level, VS/UWS patients who showed sniff response in at least one session later transitioned to MCS. Sniff response had 100% specificity and 62.5% sensitivity in predicting transition from VS/UWS to MCS. Sniff response had sensitivity of 91.7% in predicting survival after >3 y.	Yes	Recovery of consciousness (transition to MCS) and predicting survival after >3 y	Data using two different behavioral assessment protocols (this was alleviated by having a third tool applied equally to all participants). Not being able to test more frequently due to clinical schedule (possible observation of advance detection with more frequent testing). This method requires uninflated tracheostomy balloons and would not work with inflated tracheostomy balloons.
Wang et al 49 (2022)	Behavioral response to olfactory stimuli	23 7 F 16 M	22–69	1–11 mo	10 TBI 13 non-TBI	CRS-R	8 VS/UWS 15 MCS	n/a	Behavioral response to odorant stimuli compared with nonodorant stimuli (water) was higher among all patients. In response to the neutral odor presentation (1-Octen-3-ol), MCS patients had higher behavioral response compared with VS/UWS patients.	Yes	No correlation	No neuroimaging. Small sample size.
Mélotte et al 54 (2020)	Oral feeding	92 39 F 53 M	41 ± 12 VS/UWS 38 ± 12 MCS	30mo±22 for VS/UWS; 4mo±34 for MCS	60 focal 32 global	CRS-R PET	26 VS/UWS 66 MCS	PET	Presence of tracheostomy, cough reflex, and oral phase efficacy related to consciousness. 0 VS/UWS with oral feeding or efficient oral phase. 0 MCS with complete feeding.	Yes	n/a	Missing data for cough reflex criterion. Limited number of available criteria due to retrospective nature of the study.
Chatelle et al 55 (2018)	Facial expression to noxious stimuli	85 28 F 57 M	48 ± 17 VS/UWS 43 ± 17 MCS	142d, IQR: 88–396 for VS/UWS; 133d, IQR: 78–350 for MCS	35 TBI 25 ABI 25 other	CRS-R	28 VS/UWS 57 MCS	n/a	MCS had higher NCS-R scores compared with VS/UWS. Grimace observed more frequently in painful stimulation compared with nonpainful stimulation in all patients. Grimacing frequency more frequent in MCS compared with VS/UWS.	Yes	n/a	High overlap of the items in NCS-R and CRS-R. In group analyses, single CRS-R assessment was used for diagnosis. Lack of blinding, same raters carrying out both assessments.
Gélinas et al 56 (2019)	Behavioral response to noxious stimulation	147 51 F 96 M	56 ± 20	> 4 wk after brain injury	94 TBI 33 aneurysm 13 stroke 1 brain abscess	GCS	26 not conscious 56 altered 65 conscious	n/a	Higher number of active behaviors during nociceptive procedures in conscious patients. Grimace was a strong indicator for pain intensity in conscious patients.	Yes	n/a	Raters not blinded. Checklist ratings included both bedside observation and videos which may have led to differences. Only 35 patients were able to self-report their pain (limiting power of analyses).
Pincherle et al 58 (2019)	Subtle motor behavior	30 13 F 17 M	64 ± 16	10±5d	16 hemorrhage 3 metabolic 7 trauma 1 stroke 3 ABI	CRS-R MBT-r	13 coma 10 VS/UWS 5 MCS 2 eMCS	n/a	75% of coma and VS/UWS diagnosed by CRS-R showed signs of residual cognition with MBT-r. 66.7% of patients showing residual cognition by MBT-r had favorable outcome.	Yes	Yes, favorable outcome (discharge, 3 and 6 mo)	Small and heterogenous cohort.
Jöhr et al 59 (2020)	Subtle motor behavior	141 54 F 87 M	53 ± 17	35±107d for clinical CMD; 55±19d for DoC; 25±20d for non-DoC	55 severe traumatic 63 vascular 12 ABI 7 encephalopathy 4 neoplasm	CRS-R MBT-r	105 clinical CMD 19 DoC 17 non-DoC	n/a	Strong improvement trajectory of functional/cognitive recovery from admission to discharge in patients with residual cognition based on MBT-r assessment.	Yes	Yes, functional, and cognitive recovery from admission to discharge.	Categorizing patients into clinical CMD solely based on MBT-r, and not performing active mental-imagery tasks stated in the definition of CMD. No differentiation of subtypes of CMD. Potential measurement error and reliability issues due to nonstandardized assessment of outcome measures retrospectively. Medical complications and their possible impact on outcomes not taken into consideration. Data collection only at two time points, which limited information about recovery course during hospitalization.

Abbreviations: ABI, anoxic brain injury; CNC, Coma/Near Coma Scale; CMD, cognitive motor dissociation; CRS-R, Coma Recovery Scale-Revised; DoC, disorders of consciousness; DTI, diffusion tensor imaging; eMCS, emerging from minimally conscious state; F, female; fMRI, functional magnetic resonance imaging; hdEEG, high-density electroencephalography; IQR, interquartile range; M, male; MBT-r, Motor Behavioral Tool-revised; MCS, minimally conscious state; n/a, not available; NCR-R, Nociception Coma Scale-Revised; REO, resistance to eye opening; SAH, subarachnoid hemorrhage; SD, standard deviation; TBI, traumatic brain injury; VS/UWS, vegetative state/unresponsive wakefulness syndrome.

* 9 patients were discarded in data analysis due to excessive movements. The columns of etiology, diagnosis and main findings take into account only analyzed patients.

## New Potential Behavioral Signs of Consciousness in DoC Patients

### Resistance to Eye Opening

Resistance to eye opening, a firm closure of already closed eyelids when an examiner touches or tries to open the eyes, is evident in multiple neurological disorders. 31 32 33 34 The presence of resistance to eye opening and its correlation with different levels of consciousness were assessed in 79 prolonged DoC patients (TBI and non-TBI). 35 The diagnosis of patients was based on repeated CRS-R assessments. The examiners considered resistance to eye opening present when there was forceful closure of one or both eyes upon manually opening the patients' upper eyelids bilaterally. Resistance to eye opening was present in 24% of patients (19/79): 26% of VS/UWS (6/23), 53% of MCS− (8/15), and 12% of MCS+ (5/41). Although MCS+ patients had the lowest rate of resistance to eye opening, a statistically significant relationship was present between resistance to eye opening and the level of consciousness. In addition, the repeatability of resistance to eye opening was the highest in patients with MCS + , suggestive of a correlation between the level of consciousness and the number of times resistance to eye opening was seen. MCS+ patients having the lowest rate but the highest repeatability seem contradictory. One possible explanation might be that as patients recover their consciousness, they might be able to understand the instructions of the examiner and inhibit their resistance to eye opening. To replicate, validate, and better understand the relationship between resistance to eye opening and the level of consciousness, future studies could include eMCS patients and healthy controls.

Furthermore, atypical neuroimaging findings (brain activity consistent with MCS diagnosis) were more likely to be seen in VS/UWS patients with resistance to eye opening (83%) than without (29%). Indeed, five out of six patients diagnosed with VS/UWS and with resistance to eye opening had neuroimaging results more compatible with MCS. Four showed relatively preserved PET metabolism in the frontoparietal network (similar to MCS patients), and one showed response to command during a motor imagery task assessed with fMRI, suggesting that these patients were MCS*/CMD. 14 16 After 6 months of follow-up, only one of these patients showing resistance to eye opening recovered from VS/UWS, two passed away, and three remained in VS/UWS. Thus, there was no correlation between this behavior and the prognosis of these patients. Collectively, these results suggest that assessing resistance to eye opening repeatedly in prolonged DoC patients can help clinicians gain insight into patients' levels of consciousness. However, this study population had heterogeneity of etiologies and locations of brain injury. Since there might be voluntary and reflexive presentations of resistance to eye opening, future studies localizing brain lesions and correlating this with resistance to eye opening might provide more information regarding the cortical mediation of this behavior.

### Spontaneous Eye Blink Rate

Several research teams have shown that eye blink rate is modulated by fatigue, vigilance, task demand, and cognitive load. 36 37 38 To test whether there was a difference in the spontaneous eye blink rate between MCS and VS/UWS patients, 24 chronic DoC patients (TBI and non-TBI) were enrolled in a recent study. 39 Ten patients were diagnosed as VS/UWS and 14 as MCS according to the CRS-R. There were two experimental sessions for each patient, at least 24 hours apart, where patients' eye blink rate was observed at rest for 3 minutes. The examiners stood next to the patients' bed, out of patients' visual field, where they could observe and count the eye blinks. All patients were encouraged to stay relaxed with their eyes open, and not move (they were not informed about the eye blink counting to avoid potential bias). Spontaneous eye blink rate at rest (the final agreement on the rate was reached after also taking into account EEG and EOG recordings) was found to be significantly higher in MCS compared with VS/UWS patients (first session: mean of 8 ± 3 blinks for UWS and 18 ± 3 for MCS; second session: mean of 6 ± 2 blinks for UWS and 26 ± 4 for MCS patients). CRS-R index (a modified linear score taking into account the highest item in each subscale) 40 was calculated and found to significantly correlate with the mean eye blink rate at rest. Due to small sample size and fluctuations in the arousal of DoC patients in this study, more studies with larger sample sizes and more standardized assessment protocols (blinding examiners to patients' diagnosis and applying consistent timing of experimental sessions across patients) are recommended to further test the spontaneous eye blink rate as a potential indicator for the level of consciousness.

### Auditory Localization

**Fig. 3:**
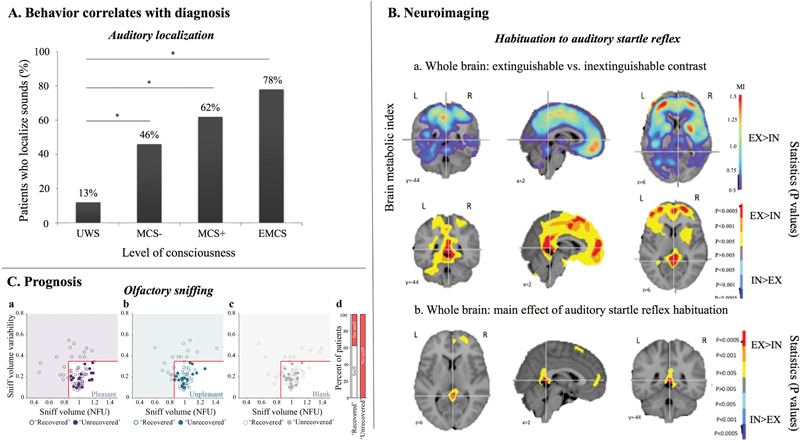
Evidence from different studies showing how behavioral, neuroimaging, and prognosis data can be used to validate signs of consciousness in disorders of consciousness (DoC) patients. ( **A** ) Behavioral results: auditory localization in DoC patients (reproduced from Carrière et al 41 ). The percentage of auditory localization increases with the level of consciousness, with significant differences between vegetative state/unresponsive wakefulness syndrome (VS/UWS) and minimally conscious state minus (MCS − ), VS/UWS and MCS + , and VS/UWS and eMCS. ( **B** ) Neuroimaging results: **a** . FDG-PET whole brain voxel-based analysis of metabolic index showing higher values in patients who had habituation to auditory startle reflex (EX) compared with patients who did not have habituation (IN), in parietal and medial frontal regions (top row), with significant differences in precuneus/posterior cingulate, premotor area, and anterior cingulate (bottom row) (adapted from Hermann et al 45 ). **b** . ANOVA showing an independent main effect of the habituation of auditory startle reflex in posterior and anterior cingulate and supplementary motor area. ( **C** ) Prognosis result: patients showing sniff response had better prognosis than patients who did not (adapted from Arzi et al 48 ): the red lines indicate a previously published sniff-response threshold, 90 dots within the boxed area (white background; bottom right) reflect sessions without a sniff response; dots outside the boxed area (shaded background) reflect sessions with a sniff response. **a** – **c** , Each dot represents a VS/UWS session. Unfilled dots indicate sessions of patients who recovered later and transitioned to MCS and filled dots indicate sessions of patients who did not recover consciousness during the study. **a** , Pleasant odorant. **b** , Unpleasant odorant. **c** , Blank. **d** , Percentage of VS/UWS patients who later transitioned to MCS (left, “Recovered”) or remain unconscious (right, “Unrecovered”) with sniff responses (white; recovered, 62.5%; unrecovered, 0%) and without sniff responses (red; recovered, 37.5%; unrecovered, 100%) across pleasant, unpleasant, and blank conditions.

In the CRS-R, spatial localization in visual and motor domains (visual pursuit and localization to noxious stimulation, respectively) is considered signs of MCS, whereas in the auditory subscale this is not the case, and localization to auditory stimulus is considered a reflex. In the CRS-R, auditory localization is evaluated by presenting auditory stimuli (patients' name, voice, noise, etc.) behind the patient, out of view, for 5 seconds twice on each side (right and left). When there is a clear head or eye movement toward the auditory stimuli on both trials in at least one direction within 10 seconds of stimulus presentation, the patient is considered to have auditory localization. 25 In a multimodal study, 186 patients with prolonged DoC (TBI and non-TBI) were assessed to examine whether auditory localization could be considered as a sign of MCS. 41 The probability of auditory localization increased with the level of consciousness: 13% of VS/UWS, 46% of MCS − , 62% of MCS + , and 78% of eMCS patients had auditory localization ( Fig. 3A ). Notably, regardless of the diagnosis, patients with auditory localization had higher survival rates after 2 years of follow-up (despite no significant differences in clinical improvement). According to the results obtained with PET, there were no significant differences in brain metabolism between VS/UWS patients with and without auditory localization. However, fMRI analysis showed higher functional connectivity between frontoparietal network and secondary occipital regions during rest in VS/UWS patients with localization compared with those without localization. High-density EEG results showed that VS/UWS patients with localization also had a higher participation coefficient in the α-band compared with VS/UWS patients without localization. The participant coefficient is a connectivity measure that has been shown to correlate with the level of consciousness in previous studies on DoC patients. 42 43 44 Taking all these results into consideration, auditory localization might be a more complex behavior than a reflex, and with the need for additional confirmation of further studies, it could be reconsidered as a potential sign of MCS.

### Habituation of Auditory Startle Reflex

In the auditory subscale of the CRS-R, auditory startle reflex is the lowest score item above zero (no response). To test its validity as a sign of MCS, habituation of auditory startle reflex was examined in 98 patients with prolonged DoC (TBI and non-TBI). 45 Habituation was assessed by presenting a loud handclap noise directly above the patients' head (out of view) 10 times consecutively (∼120 bpm), administering four trials. If patients had eyelid flutter or blink immediately after the stimulus in at least two trials, this was considered as auditory startle being present. If patients had eyelid flutter or blink after each and every clap, this was considered as inextinguishable auditory startle reflex; otherwise, patients were considered as having habituation of auditory startle reflex. Habituation was observed in 55% (53/96) of the patients. Patients who had habituation were significantly younger than patients who did not have habituation (which may have introduced a bias to the results). Habituation was present in 75% (36/48) of MCS patients, whereas it was only observed in 35% (17/48) of VS/UWS patients. Additionally, on the group level analysis, patients with habituation had higher scores in every CRS-R subscale except the communication subscale. The performance of each MCS item in the CRS-R and the habituation of auditory startle reflex in discriminating MCS patients were compared. Habituation had the highest prevalence and sensitivity, 55 and 75% [60–86], respectively, among all MCS items. The accuracy of habituation was 70%, second highest after visual pursuit (75% [65–83]). The mechanism of habituation of auditory startle reflex could be of cortical origin, as shown with FDG-PET and high-density EEG data. PET metabolic activity in multiple networks including the salience network and the default mode network correlated with the presence of habituation of auditory startle reflex ( Fig. 3B ). Higher θ- and α-power, together with higher values of cortico-cortical functional connectivity (especially, higher prefrontal–temporal connectivity), were observed in patients with habituation compared with patients without habituation. The recovery of command-following at 6 months was significantly higher in VS/UWS patients who showed habituation compared with those who did not show habituation. This new behavioral item could be considered as another sign of consciousness for MCS diagnosis and could be implemented in the CRS-R auditory scale.

### Olfactory Sniffing

The sense of smell in DoC patients has been scarcely studied; yet, accumulating findings suggest a link between olfactory abilities and the level of consciousness. A pilot study in 11 DoC patients (nine VS/UWS and two MCS) examined olfactory discrimination abilities based on patients' behavior. 46 A discriminatory olfactory response was defined by a behavioral response (eyes closure, grimace, avoiding head movement, or vocalization) to an unpleasant odor (stuffy socks-like or rancid-like) and trigeminal-irritating odor (ammonia) but not to a pleasant odor (rose-like). All MCS patients and three VS/UWS patients (33.3%) showed a discriminatory olfactory response. Notably, the three patients diagnosed with VS/UWS who showed a discriminatory olfactory response had olfactory-related activity in olfactory cortices when assessed with fMRI, suggesting that these patients might be MCS*. The six VS/UWS patients (66.7%) with no discriminatory olfactory response had no olfactory-related activity in either the primary or secondary olfactory cortices. These findings align with an fMRI study that examined olfactory neural processing in 26 VS/UWS and 7 MCS patients. 47 Odor-induced activity in primary olfactory areas was evident in 58% of VS/UWS patients (15/26) and all MCS patients (100%, 7/7). Brain activation also varied with the etiology of the lesion, where most patients with anoxic brain injury had no activation in primary olfactory areas.

A recent study investigated olfactory sniffing in 43 patients with chronic DoC (TBI and non-TBI). 48 Sensory-driven sniffing (level 1, odorant detection; level 2, odorant discrimination) reflected automatic odorant-driven response, whereas cognitive-driven sniffing reflected situational understanding and/or learning (when subjects were told they will be presented with an odorant, but they were instead presented with an empty jar; nevertheless they modified their nasal inflow). Patients were presented with pleasant (shampoo) and unpleasant (rotten fish) odors, or clean air (empty jar); their nasal inhalation volume in response to the stimuli was examined via a nasal cannula directly connected to a spirometer, and an instrumentation amplifier. Patients' level of consciousness was assessed with CRS-R and/or Coma/Near Coma Scale after each session (in addition, the Loewenstein Communication Scale was used in some cases). In total, 73 sessions were conducted in 31 MCS patients, and 73 sessions were conducted in 24 VS/UWS patients. At the group level analysis (for sessions), they observed a 10% reduction of nasal airflow from baseline in response to the odorants' presentation (regardless of pleasant or unpleasant, indicating sensory-driven level 1 sniffing response) in MCS sessions, but not in VS/UWS sessions. A similar difference was also recorded for cognitive-driven sniffing, with a 5% nasal airflow reduction in response to clean air presentation (empty jar) compared with baseline among MCS patients only. When reanalyzing the data to reflect individual level differences (of clinical importance at the patient level), sniff response had a sensitivity of 64.5% to determine MCS. Surprisingly, 10 out of 24 VS/UWS patients showed sniff response in at least one session, and all 10 patients later transitioned into MCS (during the study, 16 out of 24 VS/UWS patients transitioned to MCS). In this sample, the sniff response in VS/UWS patients therefore demonstrated 100% specificity and 62.5% sensitivity (10 out of 16 VS/UWS patients who transitioned to MCS) to predict a transition to MCS. All the patients were followed up to see how their sniff response related to long-term outcome ( Fig. 3C ). A sensitivity of 91.7% was measured for the sniff response in predicting survival at 3.1 ± 1.2 years after brain injury.

A more recent study evaluated DoC patients' behavioral responses to different olfactory stimuli, with a more qualitative approach compared with the aforementioned olfactory sniffing studies. 49 Twenty-three DoC patients (TBI and non-TBI) were enrolled in this study. Eight patients were diagnosed as VS/UWS and 15 as MCS according to repeated CRS-R. Videos were recorded while patients were being presented with one of three different olfactory stimuli: 1-Octen-3-ol (familiar neutral odor), pyridine (unpleasant fish-like smell), and water (odorless). Each odor was presented once for 3 seconds, with 15 seconds between different stimuli. The behavioral responses such as pouting, shaking head, pushing things away with hands, frowning, and twisting head in avoidance were scored by two independent and blinded (to the stimuli and diagnosis) raters. Among all patients, the behavioral responses to olfactory stimuli (1-Octen-3-ol and pyridine) were higher than nonolfactory (water) stimuli. During the familiar neutral odor session, 93% of MCS and none of the VS/UWS patients showed behavioral responses, and this difference was significant. During unpleasant fish-like smell session, 60% of MCS and 13% of VS/UWS patients showed behavioral responses, although this difference was not significant. During the odorless session, 13% of MCS and none of the VS/UWS patients showed behavioral responses. The patients were followed up after 1, 3, and 6 months with CRS-R evaluations. There was no significant correlation between behavioral response to olfactory stimuli and the prognosis.

Altogether, these findings suggest that olfactory stimuli are valuable additions to the current assessment protocols and that olfactory sniff response is a powerful and easily accessible tool which can be used in the assessment, diagnosis, and prognosis of DoC patients, further decreasing misdiagnosis rates.

### Swallowing/Oral Feeding

Previous neuropathological studies suggest that a correlation may exist between the level of consciousness and swallowing function. 50 51 52 The presence of oral feeding was investigated in 68 chronic VS/UWS patients (TBI and non-TBI) by reviewing their clinical information. These patients also underwent multimodal assessments. 53 Only 3% (2/68) of these VS/UWS patients could be fed orally. The first patient received liquid and semi liquid oral feeding in combination with gastrostomy feeding. Otorhinolaryngological exam and fiberoptic endoscopic evaluation demonstrated intact laryngeal mobility and cough reflex, and no salivary or secretions stasis. Even though no inhalation occurred, the initiation of the swallowing reflex was delayed. Clinical evaluation and neuroimaging assessments were suggestive of VS/UWS diagnosis. The second patient received full oral feeding, with solid food. Behavioral evaluations were suggestive of VS/UWS diagnosis as well, but neuroimaging and electrophysiologic assessments showed atypical findings (relative preservation of metabolism within frontal and occipital cortices, relatively preserved white matter integrity on diffusion tensor imaging, and theta activity on EEG, despite the absence of resting-state fMRI networks). Due to dissociation between behavioral and neuroimaging findings, this patient could be considered as MCS* rather than VS/UWS. This study suggests that full oral feeding and a complex oral phase of swallowing might be considered as a sign of consciousness.

A more recent study collected information regarding respiratory status, nutritional status, and otolaryngological swallowing examination from 92 patients with prolonged DoC (TBI and non-TBI). 54 Ten criteria were established: respiratory status (tracheostomy), nutritional status (feeding type), oral phase of swallowing (hypertonia of the jaw muscles, oral phase, efficacy of the oral phase), and pharyngeal phase of swallowing (pharyngo-laryngeal secretions, saliva aspiration, cough reflex, cream aspiration, liquid aspiration). The presence of a tracheostomy, cough reflex, and oral phase efficacy were found to be related to consciousness. None of the VS/UWS patients (diagnosed with multiple CRS-Rs and confirmed with hypometabolism in the frontoparietal network bilaterally using PET) had an efficient oral phase, and none could be fed orally. Additionally, none of the MCS patients received ordinary oral food. Since VS/UWS patients more frequently had a tracheostomy at the time of assessment than MCS patients, their ability to correctly manage saliva differed significantly. Taken together, these results suggested that objective and systematic assessment of swallowing should also be performed in all DoC patients, which could provide additional clinical data on the level of consciousness.

### Facial Expressions to Nociception

Pain assessment and management in DoC patients have long been an important ethical issue, since these patients cannot communicate their needs explicitly. Whether the level of responsiveness to painful stimuli could reflect the level of consciousness was investigated in a study enrolling 85 acute and prolonged DoC patients (TBI and non-TBI). 55 The levels of consciousness assessed by CRS-R total scores correlated with responses to the Nociception Coma Scale-Revised (NCS-R). Specifically, MCS patients had higher NCS-R scores compared with VS/UWS patients. CRS-R oromotor/verbal and motor subscores after noxious stimulation correlated with total NCS-R scores during noxious stimulation, and the NCS-R was not found to be more sensitive than CRS-R in assessing nociception. However, the importance of observing facial expressions to nociception was emphasized, with the results showing that grimace was observed more frequently in all patients during painful stimulation compared with nonpainful stimulation. Furthermore, there was a difference in grimacing frequency between MCS and VS/UWS patients during noxious stimulation, less frequent in the latter group. This difference may be due to the presence of tracheostomy (more frequently present in VS/UWS patients) having a possible effect on decreased lower face expression of patients. Nonetheless, observation of the facial expressions in the DoC patient population could add valuable information to the assessment.

Another study including 147 brain-injured patients (TBI and non-TBI) assessed behaviors related to standard ICU care procedures (nociceptive and non-nociceptive) in patients with different levels of consciousness. 56 Patients were classified as unconscious (GCS: 3–8), altered (GCS: 9–12), or conscious (GCS: 13–15). A behavioral checklist consisting of 30 active (grimace, tube biting, brow lowering, mouth opening, etc.) and 10 neutral (open mouth, open eyes, smile, etc.) behaviors was used for scoring. A higher number of active behaviors were observed during nociceptive procedures in conscious patients. In addition, grimace was found to be a strong indicator for pain intensity in conscious patients. The results of this study, again, emphasize that the standardized observation of facial expressions during noxious stimulation could help better classify patients.

### Subtle Motor Behavior Assessed by the Motor Behavioral Tool

The Motor Behavioral Tool-revised (MBT-r) 57 58 was developed to capture subtle motor behaviors possibly overlooked by the CRS-R. MBT-r includes seven positive signs and two negative signs ( Table 2 ). Patients were considered to have residual cognition if at least one positive item was present. The presence of a negative item suggested brainstem dysfunction and potentially abnormal automatic responses, in which case patients were not scored with MBT-r. The tool also took into consideration the inter-rater agreement of each item, eliminating reliance on an isolated item with a low inter-rater agreement. MBT-r was administered to a cohort of 30 patients with acute DoC (TBI and non-TBI) as a complementary tool to the CRS-R. 58 The authors followed up patients at discharge, after 3 months, and after 6 months, with the Glasgow Outcome Scale score, grouping them into favorable and unfavorable outcome according to their consciousness recovery (unfavorable: remaining in VS/UWS or death). Out of 24 patients classified as unconscious (coma, VS/UWS) by the CRS-R (best score out of three assessments), 18 (75%) showed signs of residual cognition with MBT-r. Also, 66.7% of patients showing residual cognition by the MBT-r had a favorable outcome. Thus, MBT-r could be a useful clinical tool to detect signs of residual cognition (subtle motor behavior) underestimated by the CRS-R, and predict recovery in acute DoC patients.

**Table 2:** The Motor Behavioral Tool-revised (MBT-r) items 58 59

The Motor Behavioral Tool-revised (MBT-r) items	
Positive signs	Spontaneous nonreflexive movements
	Response to command
	Visual fixation or visual pursuit
	Responses in a motivational context
	Defensive nonreflexive response to a noxious stimulation: nipple
	Defensive nonreflexive response to a noxious stimulation: nail bed
	Response to a noxious stimulation: grimace
Negative signs	Abnormal motor or neurovegetative responses to stimulation
	Signs of roving eyes or absence of oculocephalic reflex

Note: MBT-r consists of seven positive signs and two negative signs to assess patients with DoC for subtle motor behaviors and residual cognition.

This tool was also used in a study of 140 patients (TBI and non-TBI), where the patients were grouped into DoC (coma, VS/UWS, and MCS), non-DoC (patients who were able to interact adequately), and potential clinical CMD (patients who have residual cognition according to MBT-r assessment). 59 The latter group showed a strong improvement trajectory of functional/cognitive recovery from admission to discharge, where outcomes were measured by GOS and other outcome scales (e.g., Disability Rating Scale). 59 Collectively, these results emphasize that the combination of MBT-r and CRS-R in DoC patients could help detect covert consciousness in a substantial fraction of patients.

### Leg Crossing

Crossing legs is considered an automatic motor response in the CRS-R, one of the eleven signs that denotes MCS − . In one study, 34 patients with severe stroke who crossed their legs during their hospitalization (“crossers”) were matched with 34 severe stroke patients who did not cross their legs (“non-crossers”). 60 Patients were evaluated at admission, upon discharge, and 1 year after discharge, with GCS, NIH Stroke Scale (NIHSS), modified Rankin scale (mRS), and Barthel Index (BI). No significant differences were observed between the two groups at the time of admission, but upon discharge NIHSS and mRS were lower and BI was higher for crossers, indicating less severe neurologic deficits, less disability, and higher functional independence, respectively. After 1-year follow-up, these differences were even larger. Also, mortality was significantly lower in the “crossers” group. Leg crossing within the first 15 days after severe stroke favored better outcome in patients, and could be used as a prognostic tool. It is a sign that any healthcare provider could easily assess and needs further attention and validation by larger studies. This behavior has not been assessed in patients with DoC, but further attention is warranted in clinical practice, and further studies should assess the validity of this sign in patients with DoC.

## Discussion

In this review, we summarized recent findings regarding newly proposed behaviors denoting consciousness in patients with DoC, which could help improve the accuracy of detecting and predicting recovery of consciousness. A summary of the findings is presented in Table 1 . While they may not all reliably reflect the presence of conscious processing, the use of these behaviors can be justified based on their safe and affordable evaluation. We therefore advocate a careful observation of these new behavioral signs (resistance to eye opening, spontaneous eye blink rate, auditory localization, habituation to auditory startle reflex, olfactory sniffing, swallowing/oral feeding, facial expressions to noxious stimulation, subtle motor behavior, and leg crossing) among DoC patients when clinically appropriate ( Fig. 2 ). Raising awareness about these behaviors among caregivers, families, and all the responsible healthcare personnel might drive the development of validation studies and encourage a thorough multimodal assessment of patients presenting these clinical signs.

Beyond repeated assessments with CRS-R, we encourage the use of additional standardized tools in patients with DoC to test specific functions, such as the MBT-r or the SWADOC (SWallowing Assessment in Disorders of Consciousness—a standardized swallowing tool under validation 61 ). However, we acknowledge the challenges of time management when assessing patients in ICU settings. In this context, a new validated scale (SECONDs) has been developed to provide a faster and more practical tool to administer than the CRS-R in time-constrained clinical settings. 23 24 We also encourage continuous observation and reporting by healthcare personnel of other spontaneous motor behaviors such as tube pulling, nose scratching, and grabbing sheets. As more potential new behaviors of consciousness emerge from these observations, we recommend evaluating these behaviors in future studies in three ways: by comparing these behaviors between different states of consciousness (VS/UWS, MCS, MCS*, CMD, eMCS), by correlating each behavior with long-term outcome measures, and finally supporting these studies with neuroimaging techniques ( Fig. 3 ).

Despite established criteria for the diagnosis of MCS in standardized behavioral assessments, some of the items in the CRS-R denoting MCS are still controversial, such as visual fixation. In a study comparing the cerebral metabolism of five patients with chronic anoxic VS/UWS (therefore without visual fixation) with five patients with chronic anoxic MCS in whom the only sign of consciousness was visual fixation, no significant difference was found in cortical metabolism or cortico-cortical connectivity between the two groups. 62 Although this study used a small sample size limited to anoxic brain injury, it stresses the need to further validate some of the controversial signs of consciousness, such as visual fixation, with multimodal neuroimaging studies and larger sample sizes to elucidate the neural mechanisms supporting behavior and relate it to conscious processing.

Recent studies, beyond the scope of this article, suggest that electrophysiological findings, 63 64 65 66 67 68 69 70 71 72 73 neuroimaging findings, 16 43 74 pupil responses, 75 76 and autonomic nervous system correlates 77 78 might also reflect the level of consciousness in patients with DoC. Thus, we emphasize the need to further investigate and validate these parameters within the framework of a multimodal assessment (behavioral and neuroimaging) in DoC patients. There have been studies also suggesting that using salient stimuli, assessing behaviors within a context relevant to the patients, or performing individually tailored motivational assessments might further enhance arousal and patient participation when diagnosing patients with DoC. 59 79 80 81 82 83 84 Thus, we encourage further validation studies about the effects of implementing emotional context and salient stimuli in the assessment of DoC patients.

Regarding the diagnosis of DoC patients, some of the recommendations by the 2020 European Academy of Neurology guideline 27 are as follows: (1) passively opening patients' eyes who have no spontaneous or stimulation-triggered eye opening, and assess for both horizontal and vertical eye movements (patients with locked-in syndrome have preserved vertical eye movements) (strong recommendation); (2) using a mirror for visual pursuit, and if not elicited by a mirror, the use of pictures showing the patient's or relatives' faces or personal objects (strong recommendation); and (3) using repeated CRS-R (at least five times) assessments in the subacute–chronic setting and the Full Outline of UnResponsiveness scale in the acute setting instead of the GCS (strong recommendation). Also, the need for multicenter collaborations is highly stressed in this guideline, as well as the need for more studies investigating resistance to eye opening, 35 pupillary dilation assessment following mental arithmetic with automated pupillometry, 75 85 quantitative assessment of visual tracking, 86 87 standardized rating of spontaneous motor behavior, 58 the possibility of oral feeding, 53 evidence of circadian rhythms, 88 vegetative responses to salient stimuli, 89 and modulations of cardiac cycle (heart rate, heart rate variability, cardiac cycle phase shifts). 69 70

The diagnostic assessment of DoC patients is associated with major clinical and ethical implications. Feasible solutions to improve diagnostic accuracy are urgently needed in clinical practice, which should be addressed in a multidisciplinary approach. Further formal validation studies for the proposed new behavioral signs of consciousness summarized in this article are needed for implementation in clinical practice, although we acknowledge the challenge of lacking a gold standard reference for consciousness. Currently, very few specialized centers perform an up-to-date multimodal assessment of these patients. As a result, there is a distinct gap between scientific research and clinical practice. We encourage ICU and rehabilitation healthcare workers to collaborate with translational research teams worldwide and adopt a multidisciplinary approach in their assessment of patients with DoC, which may help bridge the gap between research and clinical practice in this field.
